# Stronger resting-state neural oscillations associated with wiser advising from the 2nd- but not the 3rd-person perspective

**DOI:** 10.1038/s41598-020-69507-9

**Published:** 2020-07-29

**Authors:** Chengli Huang, Haotian Zhang, Jinhao Huang, Cuiwen Duan, Juensung J. Kim, Michel Ferrari, Chao S. Hu

**Affiliations:** 1 Institute of Psychological Sciences, Hangzhou Normal University, 2318 Yuhang Tang Road, Hangzhou, 311121 China; 2 Art Therapy Psychological Research Centre, Hangzhou Normal University, Hangzhou, China; 3 Zhejiang Key Laboratory for Research in Assessment of Cognitive Impairments, Hangzhou Normal University, Hangzhou, China; 4 Centre for Education Studies, University of Warwick, Coventry, UK; 5 Ontario Institute for Studies in Education, University of Toronto, Toronto, Canada

**Keywords:** Neuroscience, Psychology

## Abstract

This is the first electroencephalogram study exploring the personal perspective effect on wise advising. Participants advised hypothetical protagonists in life dilemmas from both the 2nd- and 3rd-person perspective. Their advice for each dilemma was rated by two independent raters on wisdom criteria, i.e., metacognitive humility, metacognitive flexibility, and perspective taking. The results revealed that participants felt a significantly shorter psychological distance from protagonists when advising from the 2nd- (vs. the 3rd-) person perspective, *p* < 0.001. However, there was no significant effect of perspective condition on the wisdom score. Nevertheless, stronger resting-state absolute EEG powers in the frontal lobe were associated with wiser advising from the 2nd-, but not the 3rd-person perspective. Moreover, *Z* tests revealed that the correlations between the resting-state absolute EEG powers and wisdom scores were significantly stronger during advising from the 2nd- than the 3rd-person perspective. These results suggest that advising from the 2nd-person perspective was more self-related, and mental activities during rest contributed to advising from the 2nd- but not the 3rd-person perspective.

## Introduction

What makes one wise may lie in one’s resting mental activity. In fact, "resting-state" may not be resting at all. The brain is relatively active during resting-state, with an ongoing high level of neural network dynamics ready to allocate neural resources to potential brain activities^1^. When not engaged in processing external information, the resting-state brain is still undertaking internally-oriented self-related cognitive activities such as self-referential processing^2–4^, mind-wandering^5,6^, autobiographical memory retrieval and reasoning^7^, which are essential processes for wisdom derived from life narratives^8,9^. In particular, the "rest-self overlap" (i.e., the phenomenon that resting-state brain activities are always related to the processes of the internal self) may be a manifestation of wisdom from the 2nd-person perspective, which is likely to induce self-involvement. Therefore, resting-state brain activity may contribute to wisdom about life, which manifests in wise advising on others’ problems.

### Wise advising from the 2nd- versus the 3rd-person perspective

Wisdom has fascinated philosophers, theologians, and ethicists, East and West, for thousands of years^10,11^. The Berlin Wisdom Paradigm (BWP) is a well-known and well-researched open-ended performance measure of wisdom that requires participants to think aloud about brief vignettes describing difficult fictitious life problems^12^. For instance, "Somebody gets a phone call from a good friend who says that he/she can't go on any more, that she/he has decided to commit suicide. What should the person/one do and consider?"^13^. Giving wise advice (i.e., advising) for others in life dilemmas is regarded as an essential performance embodiment of "wisdom"^11–14^ in both experts and lay people’s definitions of wisdom^14–17^. Through the external behavior of advising, it is possible to gain insight into one’s wisdom. More specifically, affording flexible, context-sensitive advice reflects metacognitive humility, metacognitive flexibility, and perspective taking—three hallmarks of wisdom consistently defined by researches as "morally-grounded application of metacognition to reasoning and problem-solving"^18^.

Wisdom is not merely theoretical knowledge, but a profoundly personal phenomenon, rooted in individual experiences and the insights gained from them^19^. Therefore, self-related mental activities such as self-involvement and self-reflection should be necessary antecedents to transforming knowledge^19^ and life experience^9,20^ into wisdom. In the process of wise advising, person perspective is a subtle but important manifestation of self-involvement and self-reflection.

Most BWP studies focus on advising from the 3rd-person perspective^12^; little is known about wise advising from a 2nd-person perspective, which is common in daily life. We propose that advising from the 2nd- (vs. the 3rd-) person perspective might be more self-related. First, the 2nd- (vs. the 3rd-) person perspective should involve a smaller psychological distance from the protagonist in the hypothetical vignette, making advisers feel closer and more self-involved during advising. Psychological distance is a subjective experience that something is close or far away from the self, here, and now^21^. Calling someone "YOU" assumes that the advice-receiver is right in front of us, or at least in direct contact, whereas calling someone "HE/SHE" implies the advice-receiver is far away. Secondly, "YOU" may provide an opportunity for self-reflection. Previous studies on "self-talk" (wherein people typically refer to themselves as "You") suggest that "YOU" is expected to arise in contexts requiring explicit self-control or conscious self-guidance^22^, and plays a crucial role in introspection^23^. Moreover, generic-you, another kind of the 2nd-person perspective, is used to express more general generalizations that are deeply self-relevant and allows individuals to derive broader meanings from negative experience^24,25^. This process of meaning construction conforms to advising as expressing wisdom—through meanings derived from life narratives^26^.

Additionally, the 2nd-person perspective is also related to internally-oriented cognition, such as experience simulation^27^ and mind-reading^28,29^. While the 1st-person perspective is *subjective* and the 3rd-person perspective is *objective*, the 2nd-person perspective is *intersubjective* because it invokes a relation between an epistemic subject and another sentient being’s mental states^27,30,31^. Such an *intersubjective* perspective requires people to simulate or imagine certain experiences, as well as beliefs, desires, and emotions, to access another’s mental states^27^. Besides, unlike merely observing others from the 3rd-person perspective, the 2nd-person perspective implies interacting with others, which provides a unique kind of access to other minds^28,29^. In fact, wisdom might be a phenomenon that is more likely to be observed when multiple minds are interacting^13,32,33^. Such an interact-mind activity is well embodied in the dialogue. A previous study found that interpersonal dialogue, even imagined (internal dialogical activity), improved the level of wisdom-related performance by almost one standard deviation compared to the group which reflected on the solutions alone^13^.

To sum up, advising from the 2nd-person perspective should induce more self-related mental activities and internally-oriented cognitions, which may contribute to wise advising.

### Resting-state brain activity in the frontal lobe and its relationship with self

Spontaneous thought processes during resting-state share common brain structures with a variety of higher cognitive functions^7,34,35^. For example, the resting brain activities have been linked to the anterior prefrontal cortex^34^, which is activated during reasoning (e.g., Raven’s Progressive Matrices)^36^, and memory retrieval ^37^. The anterior prefrontal cortex is also thought to be specifically involved in the evaluation of internally generated information, or information that cannot be readily perceived from the external environment but has to be inferred or self-generated^38^. In terms of neural oscillations, resting-state electroencephalogram (EEG) reflects the electrophysiological predisposition of human behavior. For example, stronger resting-state EEG power was found to be significantly positively correlated with IQ^1,39,40^, problem-solving (e.g., anagram)^41^.

Resting-state brain activities are also related to the processes of the internal self. Previous studies found that resting-state neural activity was related to conflict-related activity in internally-guided decision making (IDM) (e.g., choosing color participants preferred), but not in externally-guided decision making (EDM) (e.g., choosing a color more frequently displayed in the experiment)^42,43^. Moreover, previous studies have demonstrated a robust neural overlap between self-related activities (e.g., label stimuli as self-referential, self-awareness, autobiographical memory) and resting-state activities within cortical midline structures (CMS)^2,3,44^, especially the anterior cortical midline structures, such as medial orbital prefrontal cortex (MOFC), medial prefrontal cortex (MPFC), ventromedial prefrontal cortex (VMPFC), pregenual anterior cingulate cortex (PACC), supragenual anterior cingulate cortex (SACC), and dorsomedial prefrontal cortex (DMPFC)^4,45–47^. Such "rest-self overlap" suggests that the brain’s resting-state activity is closely related to our sense of self, self-referential, or self-consciousness^48–50^. Further, the self-related network is also recruited during others' mind-reading, memory recall, reasoning^7^, and self-introspection^51^.

Although most "self-rest overlap" findings come from neuroimaging studies (e.g., fMRI, PET), it should be noted that hemodynamic change is significantly positively correlated with electrophysiological oscillations^52^, e.g., mean frequency of the EEG was significantly correlated with the cerebral blood flow^53^. Consistently, a growing body of EEG studies supports the interesting "self-rest overlap". For example, temporary measures of resting-state EEG as featured by temporal nestedness, temporal continuity, and temporal integration can predict self-consciousness temporally^50^. Moreover, resting-state alpha power at frontal regions before stimulus presentation or perception was significantly positively correlated with the perception of stimuli judged to be highly self-related^54^. In a cross-cultural study, higher self-referential thought (SRT) scores were associated with resting-state higher alpha activity in the anterior default mode network (ADMN) hub (i.e., superior frontal gyrus and the anterior cingulate cortex) among Taiwanese participants (but not Russian participants)^55^.

Consider together, this evidence suggests that resting-state neural activities, especially those in the frontal lobe, are associated with cognitive activities related to the inner self. Importantly, self-related cognitive activities (e.g., self-reflection and self-introspection), more easily induced from the 2nd-person perspective, are considered essential subcomponents of wisdom^29^, or for developing wisdom^17^. Thus, it can cautiously propose that resting-state brain activation should be correlated with wise advising from the 2nd-person perspective.

### Hypotheses

- The 2nd- (vs. the 3rd-) person perspective should involve a smaller psychological distance from the protagonist in the hypothetic vignette.
- Resting-state neural oscillations should be significantly positively correlated with wise advising from the 2nd-person perspective. Besides, the correlations between resting-state neural oscillations and wise advising should be stronger during advising from the 2nd- than the 3rd-person perspective.

## Materials and methods

All procedures used in the current study were approved by the ethics committee at the sponsoring University. The current study used the software program G*Power3.1 to conduct a power analysis. Our goal was to obtain 0.80 power to detect a hypothetical effect size based on the smallest effect size (*R*^2^ = 0.15) reported in previous studies on EEG correlates of self-related processing^55,56^ at the standard 0.05 alpha error probability. All methods were performed in accordance with the relevant guidelines and regulations.

### Participants

A total of 63 graduate students (28 males, 35 females; mean age 23.78 ± 1.55 years, range 19–28 years), who had never involved in BWP studies before, were recruited via campus advertisement. Twelve participants were excluded due to low voltage EEG signal or behavioral measures failure in the data analyses. Thus, the final results were based on the data of 51 participants (18 females, 33 males; mean age 23.59 ± 1.58 years, range 19–28 years). All participants were provided informed consent before the participation, and each received 80 RMB as compensation.

### Materials

#### Wisdom vignettes

Two Berlin-style fundamental life scenarios: (1) the meaning of education: "One university student is doing well in all aspects of family and study, but suddenly begins to think about the meaning of life, and then feels that it is meaningless to go to college and wants to drop out of school."; and (2) the choice between living and dying: "There is a student who grew up in a family lacked love. He does not get along well with people and has no friends. Recently, he was rejected for pursuing a female classmate and wanted to commit suicide."

Based on the traditional Berlin-style wisdom vignettes, the current study leveraged a well-adapted 2nd-person perspective wise advising paradigm in which participants imagined the lens of a camera as the eyes of hypothetical protagonists in specific life dilemmas and talked to "him"^16,57^. Previous studies showed that the paradigm had acceptable inter-rater and inter-item reliabilities, and minimized social desirability bias^57^. Different from previous research, both the 2nd- and 3rd-person perspective wise advising were included in the current study.

#### Psychological distance

The psychological distance was assessed by the Inclusion of Other in the Self (IOS) Scale^58^, which used the degree of overlap between two circles to reflect the psychological distance. Participants indicated on a 7-point Likert scale, ranging from 1 (*no overlap at all*) to 7 (*almost complete overlap*), how far do they feel from the student they helped. The higher the score, the more overlap, the smaller the psychological distance between the participant and the hypothetical protagonist.

### Procedure

The participants completed the tasks individually in a quiet laboratory room, under the guidance of a program in E-prime 2.0^59^ run on a Windows system computer. The computer screen was about 50 cm away from their faces. They seated in an armchair at a comfortable sitting position in front of a camera. A chin rest was used to help participants maintain the head position during recording.

First, the participants underwent a 6-min EEG resting-state data acquisition with eyes closed. Subsequently, the participants completed practice tasks to ensure proper use of personal pronouns and to be accustomed to the unusual situation of talking to a camera. Then, during the formal task, the hypothetical vignette and specified personal pronoun, i.e., "YOU" (the 2nd-person perspective) or "HE" (the 3rd-person perspective), were presented on the computer screen. The participants were required to close their eyes and reflect upon the vignette thoroughly with specified personal pronoun for 6 min. Afterwards, the participants were instructed to videotape 3-min advising for the hypothetical vignette by using the specified personal pronoun to address the protagonists in the vignettes. During advising, the experimenter left the room to make sure the participants felt less constrained.

The order of person perspective was counterbalanced between the participants, i.e., for each vignette, half of the participants advised from the 2nd-person perspective, and then advised again from the 3rd-person perspective; the other half participants advised from the 3rd-, and then the 2nd-person perspective. Altogether, each participant advised from the 2nd-person perspective for twice, and the 3rd- for twice (for details, see Supplementary initial instructions). After each wise advising, the participants indicated the psychological distance between themselves and the protagonist in the vignette. Finally, the participants were debriefed, compensated, and dismissed.

### Data collection and analyses

#### Wisdom ratings

Participants’ videotaped advice was transcribed. Two strictly trained undergraduate students rated participants’ advice on three wisdom criteria: metacognitive humility (MH) (i.e., recognize uncertainty and one’s limitation of knowledge), metacognitive flexibility (MF) (i.e., consider a multitude of life contexts and influencing factors, then use different rules to solve the problem/provide different advice), and perspective taking (PT) (i.e., reason about the life dilemma from others’ perspectives), which are well-acknowledged criteria for measuring wisdom^12,14,18,60^. A recent study found that the wise advising score on these three criteria could predict clinical psychologists' assessment of the potential for suicide prevention^61^. In the current study, the inter-rater reliabilities were acceptable for all wisdom criteria (*Cronbach’s Alpha* > 0.82). Therefore, the ratings were added up to get the rating on each wisdom criterion. Moreover, the inter-criteria reliabilities were acceptable for both perspectives (both *Cronbach’s Alpha* > 0.71). Therefore, the ratings on different criteria were added up to get the total wisdom score for each perspective (i.e., 2nd- or 3rd-).

#### EEG collection and processing

**Figure 1:**
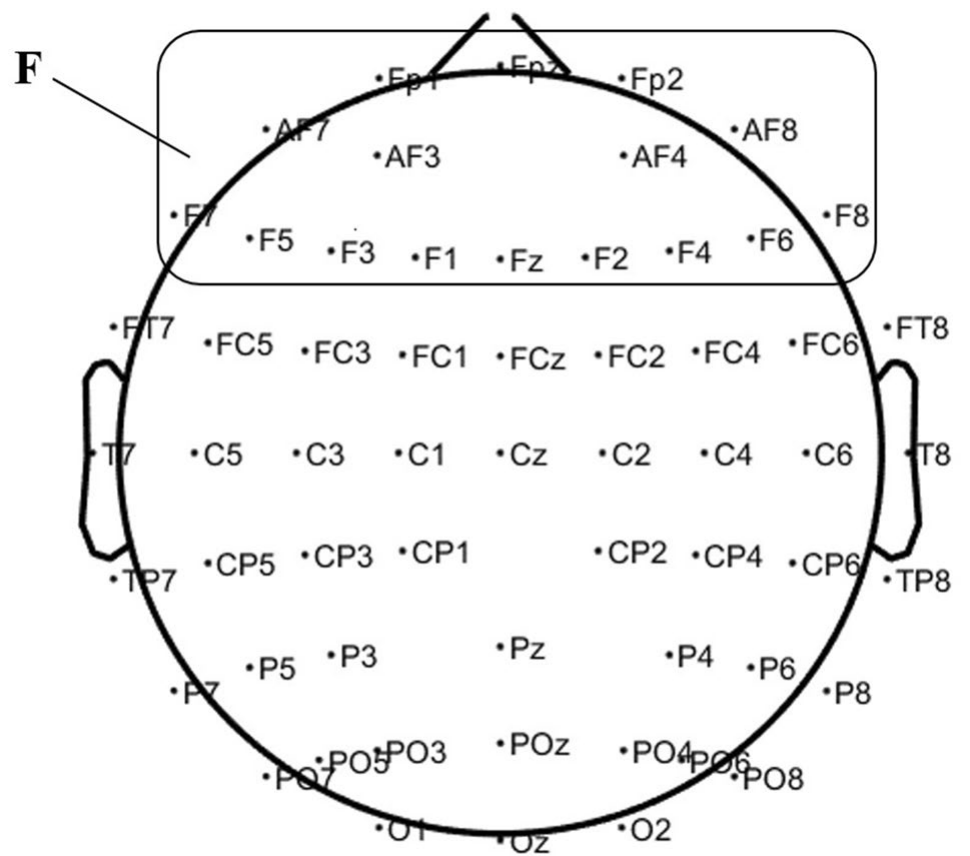
The electrocap layout and the focused area. F stands for the focused frontal lobe.

Throughout the whole experiment, EEG was recorded from 64 electrodes positioned according to the international 10–20 system and referenced to linked mastoids with a forehead ground at AFz. In the current study, we focused on the frontal lobe based on a range of sites across the scalp: Fpz, Fp1, Fp2, AF3, AF4, AF7, AF8, F1, F2, F3, F4, F5, F6, F7, F8, Fz (see Fig. 1). Electrode impedances were kept below 10 kΩ, and signals were amplified with a portable wireless EEG amplifier (NeuSen.W64, Neuracle, China) at a sampling rate of 1,000 Hz.

Data of a 6-min resting-state EEG was analyzed. During the period, the participants’ heads were fixed on a chin rest with eyes closed. EEG data were preprocessed using EEGLAB^62^, an open-source toolbox running in the MATLAB environment. Continuous EEG data were band-pass filtered (low pass: 0.01 Hz, high pass: 30 Hz). For each participant, one EEG epoch was extracted using a window-analysis time of 350,000 ms (the first 10,000 ms were not analyzed, avoiding potential distraction at the beginning of the resting or thinking stage). Trials contaminated by eye blinks and artificial movements were corrected using an Independent Component Analysis (ICA) algorithm^62^ and ADJUST, a completely automatic algorithm for artifact identification and removal in EEG data which largely matches a manual one by experts (agreement on 95.2% of the data variance)^63^. Finally, fast Fourier transformed (FFT) was applied to the processed EEG data to calculate power for different frequency bands: theta (4–7 Hz), alpha (7.5–12.5 Hz), and beta (13–30 Hz). Since the distribution of EEG power was skewed, the log transformation (log10) was applied, which produces normal distributions, according to the normality tests in SPSS 24.0.

### Data analyses

Statistical analyses were carried out in SPSS 24.0 software for Windows (version 10). Paired-sample *t* tests were conducted to test the difference in psychological distance and ratings on different wisdom criteria between the 2nd- and the 3rd-person perspective. Pearson correlational analyses (two-tail) and Spearman correlational analyses (two-tail) were conducted to analyze the correlations between individual resting-state neural oscillations (i.e., resting-state EEG powers) and wise advising from different person perspectives (i.e., scores on different wisdom criteria), depending on the distribution of data. *Z*-tests (one-tail) were conducted to test whether the correlations between the resting-state neural oscillation and wise advising were significantly stronger during the advising from the 2nd- than the 3rd-person perspective. Data were presented by means ± SD. Unless otherwise specified, significant levels were set at *p* < 0.05.

## Results

### Psychological distance

Psychological distance from the 2nd-person perspective (*M* = 4.29, *SD* = 1.48) was significantly smaller than that from the 3rd-person perspective (*M* = 3.05, *SD* = 1.36), *t* (50) = 5.90, *p* < 0.001, *Cohens’d* = 0.83, n = 51.

### Correlations between resting-state EEG powers and wisdom scores

Table 1 shows the descriptive statistics of resting-state absolute EEG powers in the frontal lobe. For the descriptive statistics of thinking-states counterpart, see Supplementary Table S1 online.

**Table 1:** Descriptive statistics of resting-state absolute EEG powers (transformed by log10) in the frontal lobe on the theta, alpha and beta band.

Resting-state absolute EEG powers			
Band	Theta M (SD)	Alpha M (SD)	Beta M (SD)
Power	6.97 (0.15)	7.32 (0.21)	7.43 (0.15)

**Figure 2:**
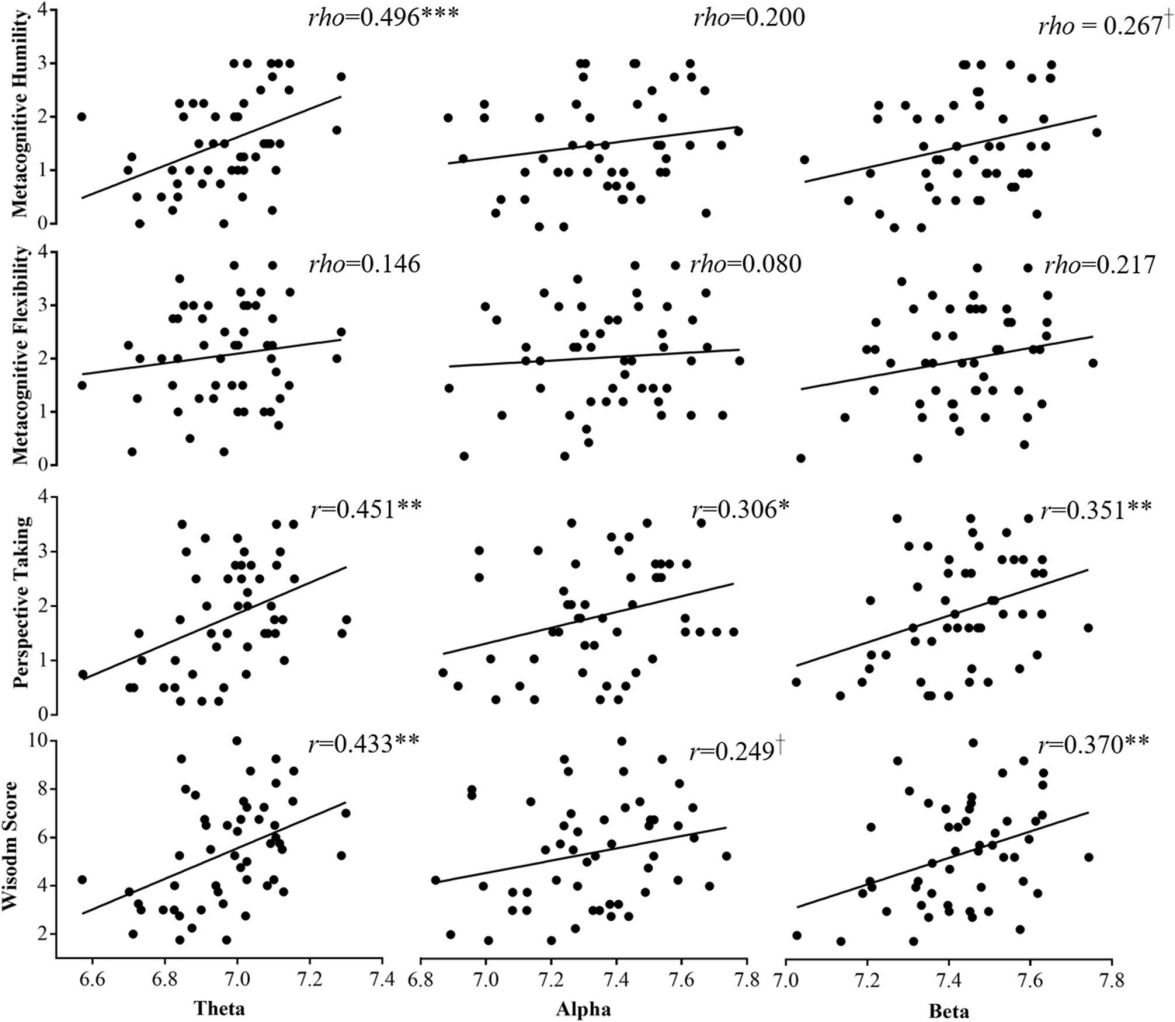
Across-participant correlations between wisdom scores from the 2nd-person perspective and resting-state absolute EEG powers in the frontal lobe. ^†^*p* < 0.1, *p < 0.05, **p < 0.01, ***p < 0.001.

**Figure 3:**
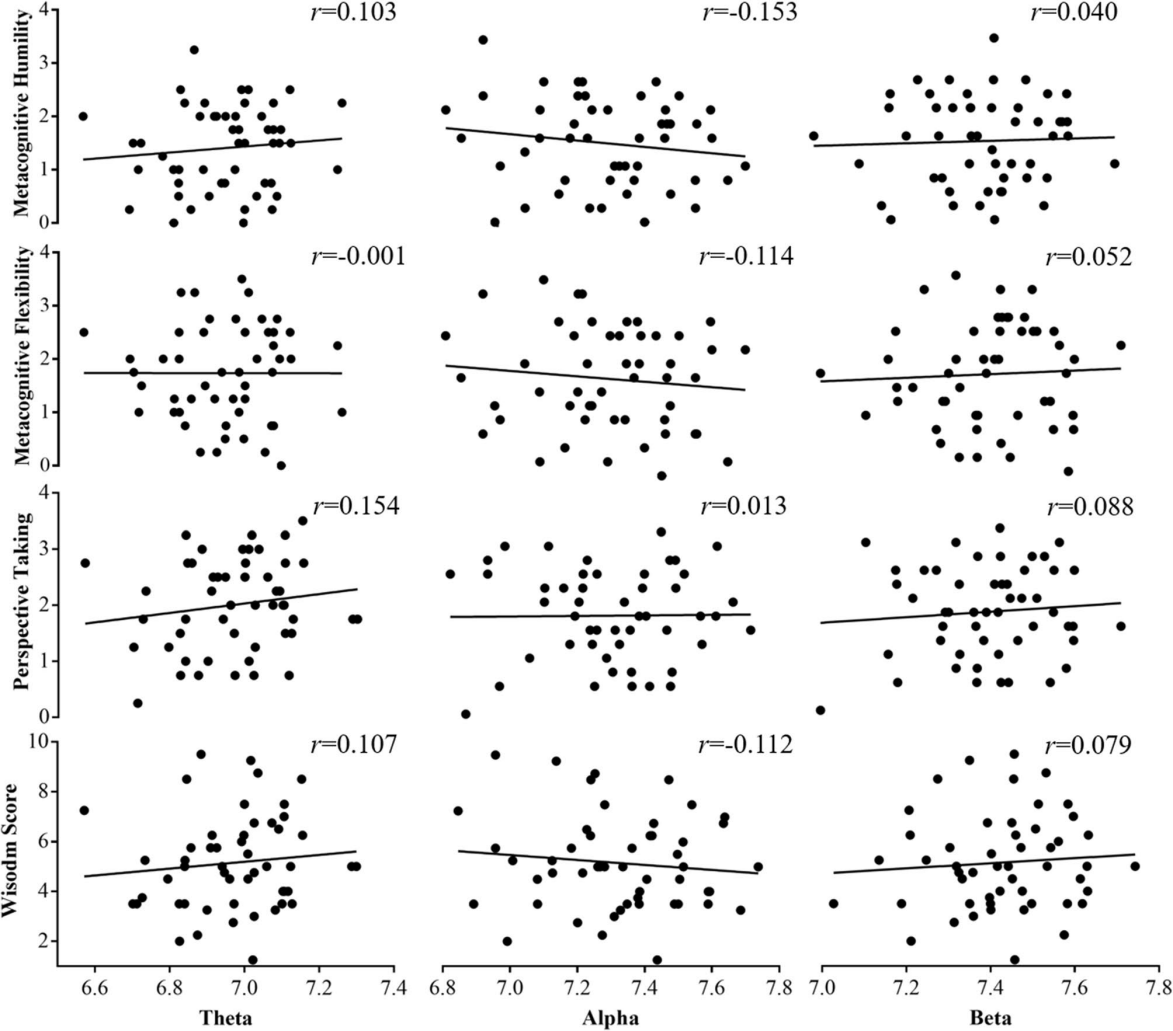
Across-participant correlations between wisdom scores from the 3rd-person perspective and resting-state absolute EEG powers in the frontal lobe. ^†^*p* < 0.1, *p < 0.05, **p < 0.01, ***p < 0.001.

Shapiro–Wilk tests indicated that most wisdom scores were normally distributed, except for metacognitive humility (MH) and Metacognitive Flexibility (MF) from the 2nd-person perspective, respectively: *p* = 0.035, n = 51; *p* = 0.026, n = 51. Therefore, Spearman correlational analyses (two-tail) were conducted to analyze the correlations between resting-state absolute EEG powers and 2nd-person-perspective MH and MF, and Pearson correlational analyses (two-tail) were conducted to analyze the correlations between resting-state absolute EEG powers and other wisdom scores. Figures 2 and 3 shows the across-participant correlations between resting-state absolute EEG powers and wisdom scores from different person perspectives. When advising from the 2nd-person perspective, the wisdom scores were significantly positively correlated with both absolute theta and beta power, respectively: *r* = 0.433, *p* = 0.001, n = 51; *r* = 0.370, *p* = 0.007, n = 51; and marginally significantly with absolute alpha power, *r* = 0.249, *p* = 0.078, n = 51. However, there was no significant correlation between any resting-state absolute EEG power and wisdom score when advising from the 3rd-person perspective, all *p*s > 0.05, n = 51.

The same Spearman correlational analyses (two-tail) and Pearson correlational analyses (two-tail) were also conducted to analyze the correlations between resting-state relative EEG powers (ratio of power in a frequency band to total power in the channel^64^) and wisdom scores from the 2nd- and 3rd-person perspective. When advising from the 2nd-person perspective, only the metacognitive humility scores was marginally significantly positively correlated with relative theta power, *r* = 0.266, *p* = 0.059, n = 51. When advising from the 3rd-person perspective, the metacognitive humility scores was significantly negatively correlated with relative alpha power, *r* = − 0.282, *p* = 0.045, n = 51; and the wisdom scores was marginally significantly negatively correlated with relative alpha power, *r* = − 0.254, *p* = 0.083, n = 51 (for the details, see Table 2).

**Table 2:** Across-participant correlations between resting-state relative EEG powers in the frontal lobe and wisdom scores from the 2nd- and 3rd-person perspective.

	The 2nd-person perspective				The 3rd-person perspective			
	Metacognitive humility	Metacognitive flexibility	Perspective taking	Wisdom	Metacognitive humility	Metacognitive flexibility	Perspective taking	Wisdom
Theta	0.266^†^	− 0.017	0.111	0.158	0.223	0.048	0.141	0.174
Alpha	− 0.009	− 0.063	0.099	0.001	− 0.282*	− 0.194	− 0.087	− 0.254^†^
Beta	− 0.018	0.174	− 0.064	0.008	0.167	0.198	0.031	0.176

^†^*p* < 0.1, *p < 0.05.

*Z* tests (one-tail) revealed that the correlations of resting-state absolute EEG powers with wise advising from the 2nd-person perspective were significantly stronger than these with wise advising from the 3rd-person perspective, especially on the theta and beta band. The same Z tests (one-tail) revealed that the correlations of resting-state relative alpha power with wise advising from the 2nd-person perspective was significantly stronger than this with wise advising from the 3rd-person perspective. To avoid an inflated Type I error rate, the alpha level was adjusted to 0.017 (0.05/3) (for the details, see Tables 3 and 4).

**Table 3:** *Z* scores for the difference in the correlations between the resting-state absolute EEG powers and wisdom scores from different perspectives.

	Theta	Alpha	Beta
Metacognitive humility	3.175**	2.666*	1.729
Metacognitive flexibility	1.132	1.491	1.281
Perspective taking	2.216*	2.084^†^	1.897^†^
Wisdom	2.980**	3.161**	2.613*

^†^*p* < 0.033, *p < 0.017, **p < 0.003.

**Table 4:** *Z* scores for the difference in the correlations between the resting-state relative EEG powers and wisdom scores from different perspectives.

	Theta	Alpha	Beta
Metacognitive humility	0.334	2.081^†^	0.105
Metacognitive flexibility	− 0.500	1.019	− 0.188
Perspective taking	− 0.210	1.295	− 0.660
Wisdom	− 0.140	2.154*	− 1.461

^†^*p* < 0.033, *p < 0.017, **p < 0.003.

We also analyzed the thinking-state neural oscillations: first, paired-sample *t*-tests revealed no significant effect of person perspective on thinking-state absolute EEG powers (see Supplementary Table S2 online); second, correlation analyses showed that thinking-state absolute EEG powers were significantly correlated with the wisdom scores when advising from the 2nd-, but not from the 3rd-person perspective (see Supplementary Table S3 online); finally, *Z* tests showed that correlations between the thinking-state absolute EEG powers and wisdom scores were significantly stronger during advising from the 2nd- than the 3rd-person perspective (see Supplementary Table S4 online). We also conducted Paired-sample *t*-tests to explore the rest-task absolute difference (subtract resting-state absolute power from the thinking-state absolute power) in the power of the respective frequencies between the 2nd-person perspective advising and the 3rd-person perspective advising. However, there was no significant difference between the two different person perspectives (see Supplementary Table S5 online).

Besides the narrow-band, rhythmic neural oscillations, the current study also explored the relationship between wisdom scores and the broadband, arrhythmic activity, indexed by the power-law exponent (PLE)^65^. The PLE was calculated using MATLAB script according to the method of a previous EEG study^50^ (the detailed script was available on: osf.io/dhpey). Unlike the narrow-band, rhythmic neural oscillations, the PLEs (*M* = 1.195, *SD* = 0.196) were not related to wisdom scores from any person perspective (for details, see Supplementary Table S6 online).

### Wisdom

Table 5 shows the descriptive statistics of wisdom scores. There was no significant difference in total wisdom scores between the 2nd- and 3rd-person perspectives, *ps* > 0.013, n = 51. To avoid an inflated Type I error rate, the alpha level was adjusted to 0.013 (0.05/4).

**Table 5:** Descriptive statistics of wisdom scores for advising from the 2nd- and 3rd-person perspective.

Criteria	The 2nd-person perspective M (SD)	The 3rd-person perspective M (SD)
Metacognitive humility	1.52 (0.87)	1.40 (0.78)
Metacognitive flexibility	2.06 (0.91)	1.75 (0.91)
Perspective taking	1.79 (0.99)	2.04 (0.80)
Total wisdom	5.38 (2.17)	5.19 (1.91)

## Discussion

The results of the present study partially supported our hypotheses. Participants reported a significantly less psychological distance from the 2nd- than the 3rd-person perspective, supporting hypothesis A. Moreover, resting-state neural oscillations were associated with wise advising from the 2nd- (not the 3rd-) person perspective, supporting hypothesis B.

The psychological distances were significantly smaller when advising from the 2nd- than 3rd-person perspective. Construal level theory (CLT) claims that the farther removed from the direct experience, the higher the level of construal (i.e., more abstract)^21^. Consequently, taking a 2nd- (vs. 3rd-) person perspective is likely to induce the participants to construct the experience of the protagonist more concretely and detailed, which is consistent with Ardelt’s (2004) interpretation of wisdom: wisdom should be personal, concrete, applied, and self-implicating rather than theoretical, abstract, and self-alienated.

There were significant positive correlations between wisdom scores from the 2nd- (not the 3rd-) person perspective and resting-state absolute EEG powers, which is widely used to explore the task-related brain activity^64,66^. As shown in Figs. 2 and 3, the resting-state absolute EEG powers in the frontal lobe were significantly positively correlated with the wisdom scores when advising from the 2nd-person perspective, but not the 3rd-person perspective. Moreover, the correlations between resting-state absolute theta and beta powers and total wisdom score were significantly stronger when advising from the 2nd-person perspective than the 3rd-person perspective. Previous studies found that skull-thickness may affect the magnitude of EEG activity^67,68^. However, the skull-thickness was not measured in the current study, and thus we cannot test this possibility.

Previous studies found that spontaneous activities of the resting-state brain share common brain structures with a variety of higher cognitive functions^7,34,35^. In particular, resting-state brain activities are relevant to self-related networks^2, 69^. Recently, a growing body of EEG studies also supports the interesting "self-rest overlap"^50,54^. Consistently, the current study found that resting-state neural oscillations were only associated with wise advising from the 2nd-person perspective when the participants felt the advice-receiver were closer to self. Previous studies on "self-talk" find that "YOU" is related to self-related mental activities^22,23^ (e.g., introspection or self-reflection). Similarly, generic-"YOU" allows people to craft a deeply self-relevant generalization, reflecting one's own negative experiences and deriving broad meanings^25^. It seems that "YOU" is a way to reflect on self and induce self-related mental activity, which is in accord with "self-rest overlap".

The "YOU"-induced self-involved mental activities probably contributed to wise advising (e.g., exploratory self-reflection), accounting for the positive correlation between neural oscillations and wisdom scores. Exploratory self-reflection is regarded as a necessary antecedent to wisdom^19,20^. Some researcher empirically examined the relationship between modes of self-reflective processing and wisdom derived from difficult life experiences^8^. They found that wisdom was positively associated with exploratory modes of processing (meaning-making, personal growth), suggesting that the development of wisdom may be partially determined by how individuals reflect upon their own significant life experiences. In terms of neurophysiology, some speculated medial prefrontal cingulate (MPFC) might be associated with instrumental or agentic self-reflection^70^. Therefore, wise advising from the 2nd-person perspective is more likely to be self-involved and may even induce exploratory self-reflection. Consistently, some participants in our study reported that advising from the 2nd-person perspective was actually like dialoguing with self. Some participants reported: "I'm also confused with the same question", "It is indeed well worthwhile to ponder the meaningless of college". These results together suggested that neural activities during rest were probably related to self-reflections that contributed to advising from the 2nd-person perspective.

## Limitation

In previous studies on "Solomon Paradox", participants reasoned more wisely from the 3rd- than the 1st-person perspective^60^. However, advising from the 1st-person perspective (advising to self) was not assessed in this study, which should be addressed in future studies.

## Conclusions

Wisdom is difficult to probe using cognitive tasks typical of cognitive neuroscience research^71^; the complicated cognitive processing associated with wisdom, therefore, increases the difficulty of fine-grained neuroscientific analyses. Nevertheless, the current study revealed that participants felt closer to the protagonist when advising about existential dilemmas from the 2nd- than the 3rd-person perspective. Such self-involvement may be the reason for the significantly stronger associations between the resting-state neural oscillations in the frontal lobe and wise advising from the 2nd- than the 3rd-person perspective. Mental activities during rest may contribute to wise advising from the 2nd- but not the 3rd-person perspective.

## Supplementary information

Supplementary Information 1.

Supplementary Information 2.

## Data Availability

The original EEG data was accessible on: https://pan.baidu.com/s/1d67peEkUTcvzl5Q0Plq_mQ; access code: adsj. The processed EEG power data, PLE script, wisdom scores, psychological distance, and wise advising transcripts (in Chinese) were accessible on: https://osf.io/dhpey/.
