# Supplementary material for: Stronger resting-state neural oscillations associated with wiser advising from the 2nd- but not the 3rd-person perspective
Source: Sci Rep. 2020 Jul 29;10:12677. doi: 10.1038/s41598-020-69507-9 (PMC7391636; doi:10.1038/s41598-020-69507-9)
Supplement: Supplementary file 1 — Supplementary Information 1. [file 41598_2020_69507_MOESM1_ESM.pdf]

# Stronger Resting-State Neural Oscillations Associated with Wiser Advising From the 2nd- but not the 3rd- Person Perspective

Chengli Huang[1,2,3], Haotian Zhang[1,2,3], Jinhao Huang[1,2,3], Cuiwen Duan[1,2,3], Juensung J. Kim[5], Michel Ferrari[5], Chao S. Hu[1,2,3*]

1 - Institute of Psychological Sciences, Hangzhou Normal University, Hangzhou, China

2 - Art Therapy Psychological Research Centre, Hangzhou Normal University, Hangzhou, China

3 - Zhejiang Key Laboratory for Research in Assessment of Cognitive Impairments, Hangzhou Normal University, Hangzhou, China

4 - Centre for Education Studies, University of Warwick, Coventry, UK

5 - Ontario Institute for Studies in Education, University of Toronto, Toronto, Canada

*Corresponding Author:

Chao S. Hu

Hangzhou Normal University, 2318 Yuhang Tang road, Hangzhou, CHINA 311121

Phone: +86 13221823963; Email: chao.hu@mail.utoronto.ca

***Intro***

We always meet some common problems during psychological counseling at school. I hope you could videotape your advice for the students who meet the problems, saying something from your heart. Maybe you could help them.

***The practice task***

A hypothetical life dilemma: “Some students think that good person often don’t get good returns, and those who pretend to be kind are more likely to succeed (in society), so they wonder if they should be good people.”

Standardized instructions guided to the participants:

> The $2^{nd}/3^{rd}$- PERSON PERSPECTIVE: “Imagine you are talking to the student’s face to face and address the student by ‘YOU’/‘HE’. Then record a three-minute video. Before recording the video, please close your eyes and think thoroughly for 6 minutes. There will be a sound to remind of you the end of the thinking time.”
>
> Note: The order of person perspective was counterbalanced between the participants.

***The experimental tasks***

Vignette of the meaning of college education (V1): "One university student is doing well in all aspects of family and study, but suddenly begins to think about the meaning of life, and then feels that it is meaningless to go to college and wants to drop out of school."

Vignette of the suicide ideation (V2): "There is a student grew up in a family lacked love. He does not get along well with people and has no friends. Recently, he was rejected for pursuing a female classmate and wanted to commit suicide."

The standardized instructions were as same as the practice task.
