# Supplementary material for: Stronger resting-state neural oscillations associated with wiser advising from the 2nd- but not the 3rd-person perspective
Source: Sci Rep. 2020 Jul 29;10:12677. doi: 10.1038/s41598-020-69507-9 (PMC7391636; doi:10.1038/s41598-020-69507-9)
Supplement: Supplementary file 2 — Supplementary Information 2. [file 41598_2020_69507_MOESM2_ESM.pdf]

Stronger Resting-State Neural Oscillations Associated with Wiser Advising From the 2nd- but not the 3rd- Person Perspective

Chengli Huang[1,2,3], Haotian Zhang[1,2,3], Jinhao Huang[1,2,3], Cuiwen Duan[4], Juensung J. Kim[5], Michel Ferrari[5], Chao S. Hu[1,2,3*]

1 - Institute of Psychological Sciences, Hangzhou Normal University, Hangzhou, China

2 - Art Therapy Psychological Research Centre, Hangzhou Normal University, Hangzhou, China

3 - Zhejiang Key Laboratory for Research in Assessment of Cognitive Impairments, Hangzhou Normal University, Hangzhou, China

4 - Centre for Education Studies, University of Warwick, Coventry, UK

5 - Ontario Institute for Studies in Education, University of Toronto, Toronto, Canada

*Corresponding Author:

Chao S. Hu

Hangzhou Normal University, 2318 Yuhang Tang road, Hangzhou, CHINA 311121

Phone: +86 13221823963; Email: chao.super.hu@gmail.com

Supplementary Table S1. Descriptive statistics for EEG power during thinking-states advising on V1 and V2 from the 2nd- and the 3rd- person perspective on the theta, alpha and beta band.

	**Theta**	**Alpha**	**Beta**
	M(*SD*)	*M*(*SD*)	*M*(*SD*)
**Vignette$_1$-perspective$_2$**	6.99(0.18)	7.31(0.22)	7.44(0.16)
**Vignette$_1$-perspective$_3$**	6.99(0.18)	7.31(0.22)	7.44(0.15)
**Vignette$_2$-perspective$_2$**	6.99(0.17)	7.31(0.22)	7.45(0.15)
**Vignette$_2$-perspective$_3$**	6.98(0.17)	7.31(0.22)	7.44(0.15)

The Paired-sample *t*-tests were conducted to explore the difference of thinking-state neural oscillations (absolute EEG powers) between the 2nd- and the 3rd- person perspectives. And the results showed that there were not significantly difference between different person perspectives on theta, alpha and beta band, respectively: $t = 0.879$, $p = 0.383$; $t = 0.029$, $p = 0.977$; $t = 1.441$, $p = 0.156$. Table S2 showed the descriptive statistics for thinking-state neural oscillations.

Supplementary Table S2. Descriptive statistics for thinking-state neural oscillations on theta, alpha and beta band for the 2nd- and the 3rd- person perspective.

Band	the 2nd-person perspective			the 3rd-person perspective		
	Theta	Alpha	Beta	Theta	Alpha	Beta
	M(SD)	*M(SD)*	*M(SD)*	*M(SD)*	*M(SD)*	*M(SD)*
Power	6.99 (0.17)	7.31 (0.22)	7.45 (0.15)	6.98 (0.17)	7.31 (0.22)	7.44 (0.15)

The Pearson correlation analyses and Spearman correlational analyses were conducted to explore the correlations between thinking-state neural oscillations (absolute EEG powers) and wisdom scores from the 2nd- and the 3rd- person perspective, depending on the distribution of data. The results were shown in Table S3. When advising from the 2nd- person perspective, the total wisdom scores were significantly positively correlated with both theta and beta absolute EEG powers, respectively: $r = 0.441$, $p = 0.001$; $r = 0.415$, $p = 0.002$; and marginally significantly with alpha absolute EEG powers, $r = 0.268$, $p = 0.057$. However, there were no significant correlations between any thinking-state absolute EEG powers and wisdom scores when advising from the 3rd- person perspective, all *p*s > 0.05.

Supplementary Table S3. Across-participant correlations (*r* or *rho* values) between thinking-state neural oscillations and wisdom scores from the 2nd- and the 3rd- person perspective.

	The 2nd- person perspective				The 3rd- person perspective			
	Metacognitive Humility	Metacognitive Flexibility	Perspective Taking	Wisdom	Metacognitive Humility	Metacognitive Flexibility	Perspective Taking	Wisdom
Theta	0.443**	0.192	0.401**	0.441**	0.157	-0.031	-0.200	0.134
Alpha	0.215	0.109	0.298*	0.268†	0.118	-0.154	0.238†	0.075
Beta	0.301*	0.299*	0.370**	0.415**	0.167	-0.078	0.112	0.078

Note: † $p < .1$, * $p < .05$, ** $p < .01$.

Z-tests (one-tail) were conducted to test whether the correlations between wise advising from the 2nd- person perspective and the counterpart thinking-state neural oscillations (absolute EEG powers) were significantly stronger than these between wise advising from the 3rd- person perspective and the counterpart thinking-state neural oscillations (absolute EEG powers). The results showed that the correlations between the thinking-state absolute EEG powers and wisdom scores were significantly stronger during advising from the 2nd- than the 3rd- person perspective on the theta and beta band, respectively: $Z = 1.659$, $p = 0.049$; $Z = 1.781$, $p = 0.037$.

Supplementary Table S4. Z scores for the difference in the correlation of thinking-state neural oscillations with wisdom scores of advising between different person perspectives (i.e., the 2nd- person perspective and the 3rd- person perspective).

	Theta	Alpha	Beta
Metacognitive Humility	1.556ⱡ	0.489	0.696
Metacognitive Flexibility	1.104	1.297ⱡ	1.894*
Perspective Taking	1.088	0.317	1.352ⱡ
Wisdom	1.659*	0.978	1.781*

Note: ⱡ $p < .1$, * $p < .05$

The Paired-sample *t*-tests were conducted to compare differences in changed rest-think absolute EEG power (subtract resting-state absolute power from the thinking-state absolute power) between the 2nd- and the 3rd- person perspective. And the results showed that there was no significant difference on changed rest-think absolute EEG power between the two different person perspectives on theta, alpha or beta band, respectively: $t = 0.879$, $p = 0.383$; $t = 0.029$, $p = 0.977$; $t = 1.441$, $p = 0.156$. Table S5 showed the descriptive statistics for changed rest-think absolute EEG power.

Supplementary Table S5. Descriptive statistics for rest-think absolute EEG power on theta, alpha and beta band for the 2nd- and the 3rd- person perspective.

Band	the 2nd-person perspective			the 3rd-person perspective		
	Theta	Alpha	Beta	Theta	Alpha	Beta
	M(SD)	*M(SD)*	*M(SD)*	*M(SD)*	*M(SD)*	*M(SD)*
Power	0.018 (0.07)	-0.015 (0.06)	0.020(0.06)	0.013 (0.06)	-0.015 (0.05)	0.013 (0.04)

The Pearson correlation analyses and Spearman correlational analyses (depending on the distribution of data) were conducted to explore the correlations between power-law exponents and wisdom scores from the 2nd- and the 3rd- person perspective. And the results showed that power-law exponents were related to no wisdom score for any person perspective, all *p*s > 0.05.

Table S6. Across-participant correlations (*r* or *rho* values) between power-law exponents and wisdom scores from the 2nd- and 3rd- person perspective.

	The 2nd- person perspective				The 3rd- person perspective			
	Metacognitive Humility	Metacognitive Flexibility	Perspective Taking	Wisdom	Metacognitive Humility	Metacognitive Flexibility	Perspective Taking	Wisdom
PLE	0.251ⱡ	-0.052	0.111	0.098	0.115	-0.077	0.065	0.038

(*Note*: ⱡ *p* < .1).
